# Intrasexual selection: Kin competition increases male‐male territorial aggression in a monogamous cichlid fish

**DOI:** 10.1002/ece3.6759

**Published:** 2020-09-12

**Authors:** Simon Vitt, Jenny Hiller, Timo Thünken

**Affiliations:** ^1^ Institute for Evolutionary Biology and Ecology University of Bonn Bonn Germany

**Keywords:** inclusive fitness, intrasexual competition, kin avoidance, kin recognition, sexual selection

## Abstract

During intrasexual competition, individuals of the same sex compete for access to breeding sites and mating partners, often accompanied by aggressive behavior. Kin selection theory predicts different kin‐directed social interactions ranging from cooperation to aggression depending on the context and the resource in question. Kin competition reducing indirect fitness might be avoided by actively expelling relatives from territories and by showing higher aggression against kin. The West‐African cichlid *Pelvicachromis taeniatus* is a monogamous cave breeder with males occupying and defending breeding sites against rivals. This species is capable of kin recognition and shows kin‐preference during juvenile shoaling and mate choice. However, subadults of *P. taeniatus* seem to avoid the proximity of same‐sex kin. In the present study, we examined territorial aggression of territory holders against intruding related and unrelated males as well as intruder's behavior. We observed higher aggression among related competitors suggesting that related males are less tolerated as neighbors. Avoidance of intrasexual competition with relatives might increase indirect fitness of males in monogamous species.

## Graphical abstract

Kinship promotes intrasexual aggression in a territorial context. Confronting male territory holders with related and unrelated intruders results in higher aggression when the intruders are related. Expulsion of related rivals may ultimately lead to reduced intrasexual kin competition over mating partners.

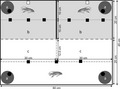

## 1 INTRODUCTION

Intrasexual competition is a major force driving contest behavior, morphology, and ornament evolution in males (Andersson, 1994; Clutton‐Brock, 2007). Access to breeding sites and females (Andersson, 1994) and the defense of established territories are often mediated by aggressive behavior of males exercised against rivals. Intrasexual aggression can have severe consequences and high costs not only for competing males, for example, in form of injuries and stress (e.g., Karino & Someya, 2007), but also for the female partner that may suffer from mating with aggressive males (Rankin, Dieckmann, & Kokko, 2011) increasing sexual conflict (reviewed in Pizzari & Gardner, 2012).

Kin selection theory (inclusive fitness theory) predicts that interactions between genetic relatives can alter inclusive fitness by affecting individual's indirect fitness (Hamilton, 1964). Inclusive fitness was defined by Griffin and West (2002) as “the effect of one individual's actions on everybody's numbers of offspring weighted by the relatedness” (p. 20) including the individual's personal offspring (direct fitness component). The indirect fitness is a component of the inclusive fitness that refers only to the effect of one individual's actions on other's number of offspring devalued by the coefficient of relatedness (Grafen, 1982; Lucas, Creel, & Waser, 1996; Oli, 2003).

Kin‐biased interactions can be formed by two mechanisms: Kin discrimination and limited dispersal (Cornwallis, West, & Griffin, 2009). Kin discrimination, that is, the differential treatment of kin and nonkin based on kin recognition (the cognitive ability to differentiate between related and unrelated individuals (Hepper, 2005; Mateo, 2004; Penn & Frommen, 2010)) is widespread in animals. Kin discrimination is relevant in diverse contexts ranging from mate choice (reviewed in Szulkin, Stopher, Pemberton, & Reid, 2013), grouping decisions to cooperation and aggression in groups (Brown & Brown, 1993a, 1993b; Edenbrow & Croft, 2012; Griffiths & Armstrong, 2002; Hesse, Anaya‐Rojas, Frommen, & Thünken, 2015; Makowicz, Moore, & Schlupp, 2018; Thünken, Hesse, Bakker, & Baldauf, 2015). Generally, kin discrimination in intrasexual selection has received less attention and, in particular, experimental evidence for higher aggression against related competitors is scarce.

Limited dispersal in “viscous populations,” or patch‐structured populations can also lead to more frequent interactions between relatives without kin recognition which may promote the evolution of altruistic behavior, but also promotes competition for resources between relatives which balances one another (Taylor, 1992; but see Whitlock & Van Dyken, 2011). In theory, patches of altruism in purely viscous populations are prone to be invaded by selfish types of neighboring patches (Wilson, Pollock, & Dugatkin, 1992). However, in populations which are not purely viscous, that is, by having a dispersal stage, individuals may benefit from competing against nonrelatives which, in turn, may result in such viscosity promoting the evolution of altruism (Queller, 1992). In addition, theory predicts that the availability of reproductive resources for each breeder, the heterogeneity in patch quality, also mediates social evolution (Rodrigues & Gardner, 2012).

While the key role of relatedness in social evolution is well‐established (Griffin & West, 2002), its impact on inter‐ and intrasexual selection is less examined. Kin competition can decrease (e.g., Gandon, 1999) and kin cooperation can increase indirect fitness (West, Pen, & Griffin, 2002). Kin avoidance may occur in form of dispersal behavior which has been predicted to be an important mechanism to reduce competition among kin (Greenwood, 1980; Hamilton & May, 1977) which was confirmed in recent studies (Bitume et al., 2013; Bonte et al., 2012; Innocent, Abe, West, & Reece, 2010). Such kin‐biased social behavior often is context dependent and can be contrary between different time points. Whereas groups of juvenile Atlantic salmon (*Salmo salar*) show reduced aggression and lower distances to nearest neighbor compared with nonkin groups during growth phase in summer (Brown & Brown, 1993b), kin‐directed behavior was reversed during winter in this species, expressed by active avoidance of kin when sheltering (Griffiths, Armstrong, & Metcalfe, 2003).

The relative importance of kin cooperation and kin competition is often affected by the mating system and environmental conditions. In male wild turkeys, indirect fitness benefits of cooperation between relatives during courtship offsets the costs for helpers giving an explanation for the evolution of such cooperative behavior (Krakauer, 2005). Kin competition is supposed to affect the relation between mating system and sex‐biased dispersal. Moreover, simulations suggest that kin competition promotes dispersal of one sex under monandry and polyandry, but should be balanced under monogamy (Brom, Massot, Legendre, & Laloi, 2016).

Leaving familiar environments is risky and often linked to high migration costs, e.g. by enhanced predation risk (Cote, Fogarty, Tymen, Sih, & Brodin, 2013; Yoder, Marschall, & Swanson, 2004) or energy consumption (Bonte et al., 2012). Therefore, plastic kin avoidance dependent on the risk or intensity of kin competition should be favored by selection (Van Petegem et al., 2018; Vitt, Madge Pimentel, & Thünken, 2020). Kin‐directed aggressive behavior aiming to actively expel related conspecifics out of the territory could be an alternative mechanism to reduce kin competition. Forcing potential competitors to leave the natal area have been shown in field studies with birds (Aguillon & Duckworth, 2015; Strickland, 1991), and in banded mongooses (*Mungos mungo*) it was shown that dominant individuals specifically force closely related females to leave the group (Thompson et al., 2017).

In the present study, we examined the effect of kinship on territorial aggression in male *Pelvicachromis taeniatus,* a monogamous cichlid fish (Langen, Thünken, & Bakker, 2013) with mutual mate choice and biparental brood care (Thünken, Bakker, Baldauf, & Kullmann, 2007a). When becoming reproductively active, males occupy territories, including for example, caves as suitable spawning site (Thünken et al., 2011), which are defended against rivals or intruders. Females compete among each other for access to males holding a territory (Baldauf, Bakker, Kullmann, & Thünken, 2011; Thünken, Bakker, Baldauf, & Kullmann, 2007b). Kin discrimination was examined multiple times in *P. taeniatus* and is expressed during shoaling (Hesse & Thünken, 2014; Thünken et al., 2015), cooperation (Hesse et al., 2015) and mate choice with preference for kin (Thünken et al., 2007a, 2007b; Thünken, Meuthen, Bakker, & Baldauf, 2012).

This study uses adult male *P. taeniatus* to explore the effect of kinship on male‐male territorial aggression and competition during the establishment of breeding sites. In this experiment, two unrelated territory holding males, which were visually separated from each other, were exposed to a same‐sex intruder, which was related (full‐sibling) to one of the territory holders. Kinship can promote cooperation (West & Gardner, 2010) which, on the one hand, may lead to individuals showing tolerance to the presence of kin, that is, less aggression, as they potentially could cooperate when defending breeding sites against predators. This could, however, lead to increased competition with kin over resources, e.g. mating partners, which should be avoided (Hamilton & May, 1977; Queller, 1992; West et al., 2002). Dispersal can reduce competition between neighbors as it shifts competition from being local to being global (Lehmann & Rousset, 2010). In the present study, emigration, that is, dispersal, was impossible. However, when relatives are not tolerated as neighbors, kin‐promoted aggression during territory establishment may lead to lower direct competition with kin over mating partners.

## 2 METHODS

### 2.1 Experimental animals

The experimental animals used in this study were adult males from the third generation of laboratory bred *Pelvicachromis taeniatus*, descending from individuals caught in the Moliwe River (near Limbe, West Cameroon 04°040N/09°160E). Individuals were obtained from a captive breeding at the Institute for Evolutionary Biology and Ecology of the University of Bonn (see Thünken et al., 2007a). After spawning, clutches of eggs were removed from the parents and placed in 1 L plastic boxes equipped with an airstone. At an age of one month, sibling‐groups were split into two groups and transferred to larger tanks, measuring 30 × 20 × 20 cm (length × width × height), including sand as substrate, Java moss (*Vesicularia dubyana*) as shelter and Tetra Brillant Filters (Tetra Co. Ltd, Japan). After two more months, fish were transferred into tanks measuring 60 × 40 × 30 cm (length × width × height), also equipped with sand, Java moss and a filter (model: Gully filter; Hobby, Germany). During the first two months, fish were fed *ad libitum* daily with *Artemia* nauplii. From the third month on, individuals were now fed with a mixture of defrosted adult *Artemia* and mosquito larvae. During rearing, groups were visually isolated from each other by opaque plastic sheets, the water temperature was kept at 25 ± 2°C and the light‐dark cycle was set to 12:12 hr light:dark cycle.

In total, 40 trials were conducted using 120 adult male *P. taeniatus* from 11 families. Forty individuals served as intruders and 80 were used as territory holders. When being tested, intruders had an age of 482 ± 76 (mean ± *SE*) and territory holders 471 ± 53 (mean ± *SE*) days. Standard length was measured to the nearest millimeter using a measuring board with scale paper (intruder: 5.92 ± 0.05, TH: 5.97 ± 0.04, mean ± *SE*). Experiments were conducted in October 2018.

### 2.2 Experimental setup

**FIGURE 1:**
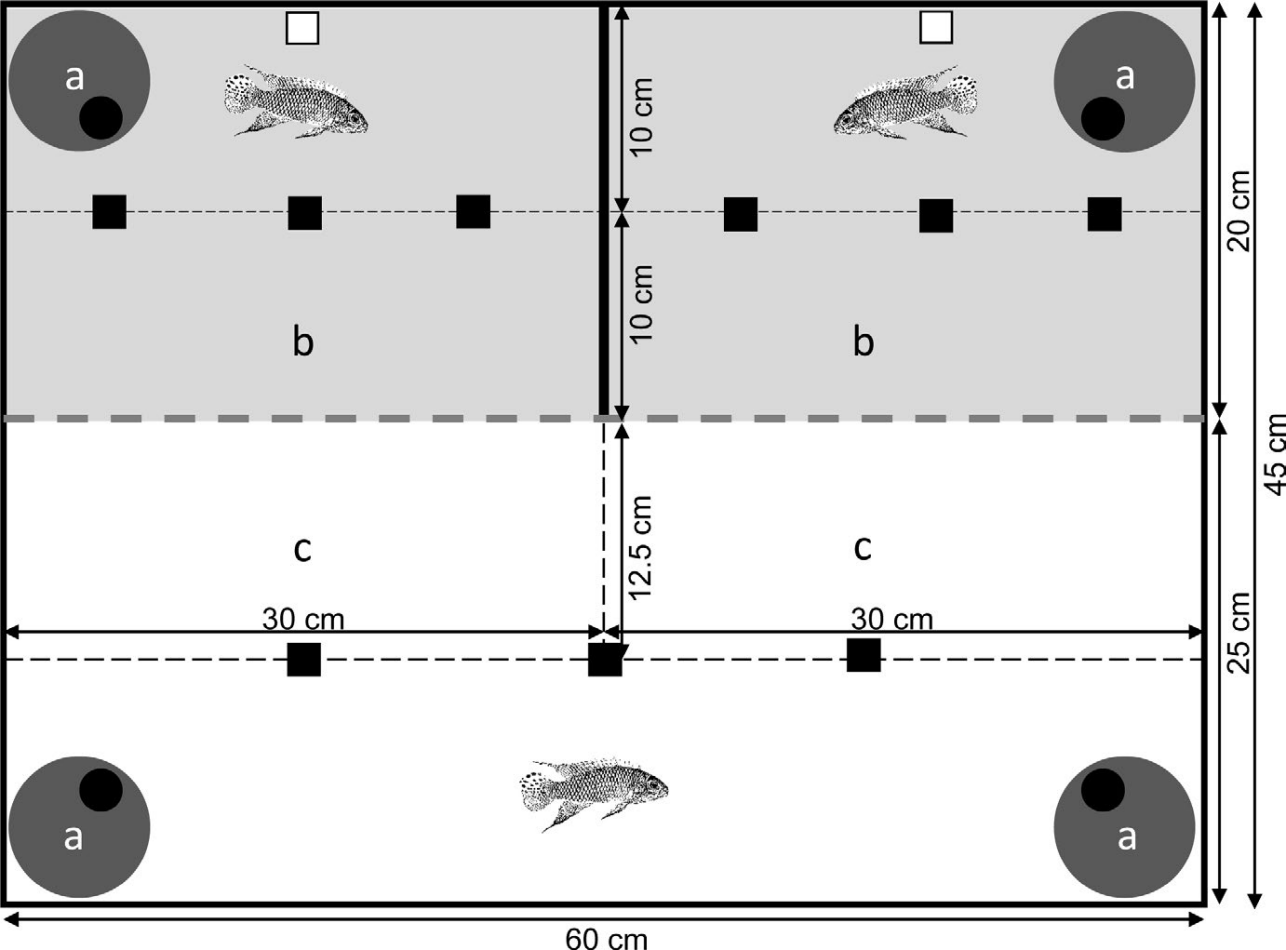
Experimental setup: The experimental tank was subdivided by a transparent, perforated sheet of plexiglas (gray, dashed line) into a territory holder area (gray) and an intruder area (white). Within the territory holder area, an opaque sheet of gray plastic created two compartments, each including a territory holder, a cave (a) and an airstone (white square). The association zones of the territory holders (b) and intruder (c) were marked in each compartment by three cubic stones (black squares)

The experimental setup consisted of six experimental tanks which allowed six trials to be conducted simultaneously. Each tank measured 60 × 45 × 30 cm (length × width × height), including sand as substrate and filled up to a water level of 15 cm (Figure 1). The tanks were subdivided into two compartments using a perforated and transparent sheet of plastic. One side served as intruder compartment, measuring 60 × 25 cm (length × width) and the other one as compartment for the territory holders, measuring 60 × 20 cm (length × width). By placing an opaque sheet of plastic within the territory holders’ compartment, two separate areas were created, each for one male, measuring 20 × 30 cm (length × width). In all four corners of each experimental tank, a ceramic cave was placed. Therefore, each of the territory holder areas contained one cave and the intruder compartment contained two caves. Furthermore, each of the territory holder areas was equipped with an airstone ensuring sufficient oxygen supply. Within the intruder compartment, two zones, each measuring 30 × 12.5 cm (length × width), were marked close to the territory holder compartment by placing three small stones centered, lengthways as markers. This was also done within both territory holder areas, creating zones close to the intruder compartment, measuring 10 × 15 cm (length × width) each.

During an experimental trial, a camera (Logitech QuickCam Pro 9000) connected to a laptop (Fujitsu Lifebook S Series) was placed in a distance of 40 cm from diagonally above to the intruder compartment. Both sides and the back of each tank were covered with opaque sheets of plastic to avoid external disturbances. The experimental setup was designed to allow the intruder to choose between potential territories close to kin or nonkin conspecifics while preventing visual communication between both territory holders. During the experimental period, fish were fed with mosquito larvae daily *ad libitum*, light was provided in a 12:12 hr light:dark cycle, by a fluorescent tube (Osram L36 W/11‐860, Lumilux Plus Eco) and the water temperature was kept at 24 ± 1°C.

### 2.3 Experimental procedure

Two territory holders were separately transferred to each experimental tank using a 1‐L plastic box. One territory holder was carefully placed by hand in each of the two areas within the territory holder compartment. An experimental trial was only started when both males had accepted their compartment, including the cave, as their territory. Therefore, they were given two days to acclimatize and occupy this area. Individuals were assumed to be acclimatized when they entered the cave regularly and built a mound of sand in front the cave, which represents a part of establishing a breeding cave. Experiments were conducted in a paired design, that is, a pair of territory holders was used for two trials. In the first trial, the intruder was unfamiliar kin of the one territory holder and in the second trial of the other territory holder. Between the two trials, there was at least one day break to reduce influences of olfactory cues between intruders. In total, 17 paired (17 territory holders, used twice and 34 intruders used once) and 6 unpaired (territory holders and intruders used once) trials were conducted, due to the absence of appropriate fish serving as territory holder, during six trials.

After the start of the video recording, an intruder was carefully transferred from the group tank to the experimental tank and placed by hand in the center of the intruder compartment. After six hours (1 p.m.–7 p.m.), the recording was stopped automatically and was continued the next day for 3 hr (9 a.m.–12 p.m.). After each trial, the standard lengths of the males were measured (SL_intruder_ and SL_TH_: snout to the beginning of the tail fin) to an accuracy of 1 mm using scale paper and the differences in size between intruder and the two territory holders calculated. Additionally, size differences in length between kin (SL_kin_) and nonkin (SL_nonkin_) territory holders were calculated (SLdiff_kin‐nonkin_) and size differences between intruder and kin (SLdiff_kin‐intruder_) as well as intruder and nonkin (SLdiff_nonkin‐intruder_).

### 2.4 Video analysis

Video recordings were analyzed using the Behavioral Observation Research Interactive Software (BORIS, v.6.2.2) (Friard & Gamba, 2016). Time close to kin and nonkin and the number of approaches to kin and nonkin were measured for the intruder and territory holders as a measurement for restrained aggression display as in this species interactions between two reproductive active males could always be interpreted in an aggressive context as cooperation between adult males is absent. Furthermore, times the intruder spent in the cave close to kin and nonkin were measured. Additionally, aggressive behavior outgoing from the intruder directed to kin and nonkin as well expressed by both territory holders to kin and nonkin were quantified. Aggression was defined as a fast approach toward the conspecific combined with an attempted bite.

The videos were analyzed from the timepoint on when the intruder entered the zone close to a territory holder for the first time and were stopped after two hours. Exceptions were made if the intruder showed a startle response when being placed in the experimental tank, that is, entered the zone during a panic reaction, which was defined as random and rapid swimming across the compartment. In that case, analyses were started after the intruder entered the zone a second time while showing normal swimming behavior.

An index was calculated for the visits of the intruder to kin or nonkin conspecifics by subtracting visits to nonkin from those to kin (visits‐intruder_index_). Analogous, an index was calculated for the times the intruder spent close to kin or nonkin by subtracting times spent close to nonkin from times spent close to kin (time‐intruder_index_). Furthermore, indices were calculated which show the relative proportion (%) of visits and times from territory holders directed to kin and nonkin (visits‐TH_rel_, time‐TH_rel_).

### 2.5 Statistical analysis

Statistical analysis was performed with R, version 3.5.2. (R Core Team, 2019). The first choice of the intruder (zone close to kin or nonkin) was analyzed using a binomial exact test. For further analyzes, normal distribution was tested using the Kolmogorov–Smirnov test with Lilliefors correction. Variables visits‐intruder_index_, time‐intruder_index_, visits‐TH_rel_, and time‐TH_rel_ met the assumptions of normality and were analyzed by conducting linear mixed‐effects models (LME) using the lme4‐package (Bates, Maechler, Bolker, & Walker, 2014). For analyzing intruder behavior, LMEs including the dependent variables visits‐intruder_index_ and time‐intruder_index_ and the explanatory variables SL_intruder_, SLdiff_kin‐nonkin_ were conducted. Furthermore, the deviation from zero (no difference) was analyzed by the Intercept of the best explaining model. As random factors, the territory holder pair together with the family of the intruder were included in the LMEs. For analyzing territory holder behavior, the dependent variables visits‐TH_rel_, time‐TH_rel_ together with the explanatory variables SL_intruder_, SLdiff_TH‐intruder_, number of attacks and kinship were included in the LMEs. As random factors, trial number together with family of the territory holders were included in each model. Nonsignificant variables were removed stepwise from the LMEs in the order of their statistical relevance using the backward elimination procedure of the “step” function in the lmerTest package (Kuznetsova, Brockhoff, & Christensen, 2017). Significance values for the fixed effects were based on F‐tests with Kenward‐Roger approximation. Variables attacks of intruder directed to territory holders as well as attacks of territory holders directed to intruders differed significantly from normal distribution and failed to respond to any transformation. Moreover, due to over‐dispersion, generalized linear mixed‐effects models could not be applied. Thus, to test whether the number of attacks from intruder and territory holders directed to kin or nonkin differed, paired Wilcoxon‐Tests were applied.

## 3 ETHICS

All animal procedures complied with the European Directive (2010/63/EU) on the protection of animals used for experimental and other scientific purposes.

## 4 RESULTS

**FIGURE 2:**
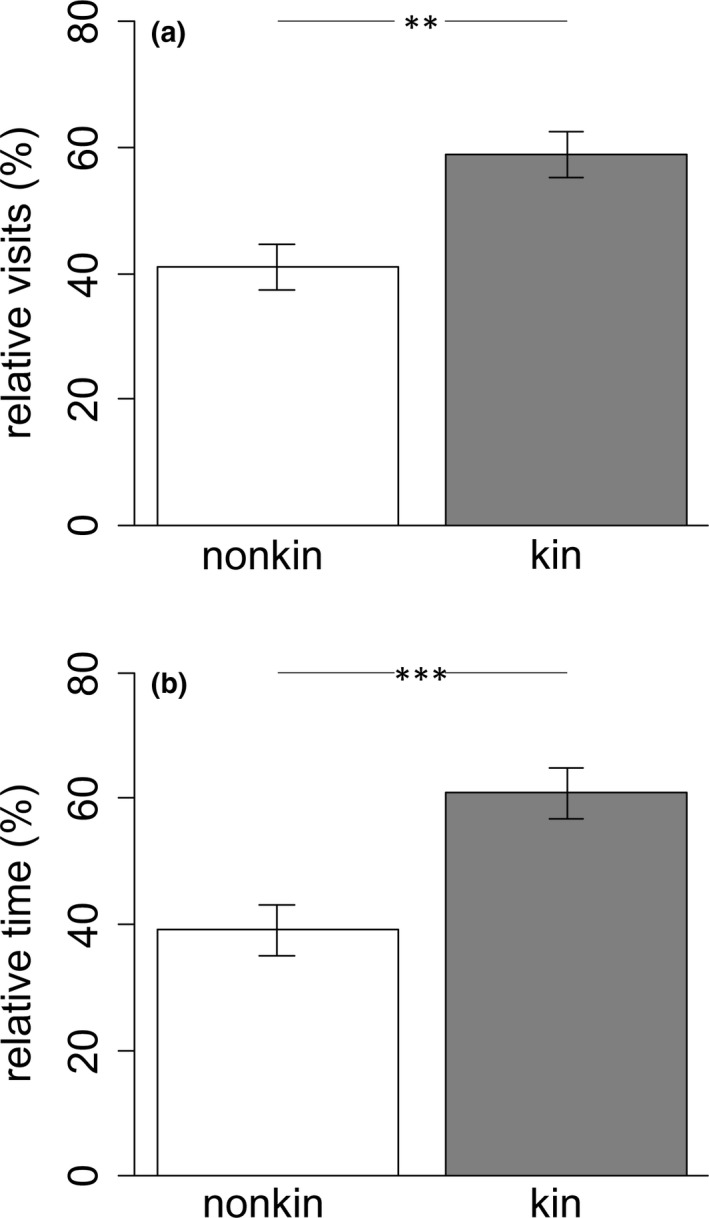
Shown are the indices of (a) visits to and (b) times spent in the preference zone close to nonkin (white) and kin (gray). Given are means and *SE*. **p < .01, ***p < .001

The intruders did not significantly differentiate between kin or nonkin during their first approach (kin/nonkin: 21/19, binomial test, *p* = .875). The relative visits as well as the relative times the territory holders spent close to kin or nonkin differed significantly and were higher for zones close to kin (Table 1, visits: Figure 2a, times: Figure 2b). In addition, there was a positive relationship between the dependent variable times spent close to kin or nonkin and the explanatory variable number of attacks (Table 1). Standard lengths of territory holders, intruders, and the differences between both lengths had no significant impact on the observed territory holder behavior (Table 1).

**TABLE 1:** All linear mixed‐effects models calculated of the effects of kinship and body traits on the indices regarding visits (visits‐intruder_index_) and times (time‐intruder_index_) for the intruders and regarding the proportion (%) of visits (visits‐TH_rel_) and times (time‐TH_rel_) for the territory holders

Dependent variable	Explanatory variables	*F*‐value	*t*‐Value	*p*	Random factors
visits‐intruder_index_	SL_intruder_	5.332		**.026**	Territory holder‐pair family of intruder
	SLdiff_kin‐nonkin_	8.349		**.006**	
	Intercept		−2.211	**.033**	
time‐intruder_index_	SLdiff_kin‐nonkin_	0.103		.750	
	SL_intruder_	0.683		.413	
	Intercept		1.412	.206	
visits‐TH_rel_	Attacks	0.022		.884	Trial family of territory holders
	SL_intruder_	0.131		.719	
	SLdiff_TH‐intruder_	0.134		.715	
	kinship	11.600		**.001**	
time‐TH_rel_	SLdiff_TH‐intruder_	0.237		.628	
	SL_intruder_	1.560		.215	
	Attacks	10.956		**.001**	
	kinship	13.797		**<.001**	

Note

For detailed explanation of variables, see the text. During stepwise model reduction, degrees of freedom always differed by one. Significant results are printed in bold (*p* < .05)

The index of relative visits (visits_index_) differed significantly from chance (50%) (Table 1) representing more frequent approaches toward related conspecifics. The difference between standard length of the intruder and its related conspecific (SLdiff_kin‐intruder_) and the index of relative visits correlated negatively (Table 1), while there was no significant relationship between standard length difference of intruder and unrelated territory holder (SLdiff_nonkin‐intruder_) (Table 1). In addition, standard length of the intruder and the difference in standard length between both territory holders also had no significant effect on the index of relative visits (Table 1).

The index of relative times which the intruder spent close to either related or unrelated conspecifics did not differ from chance (Table 1) and was not affected by standard length differences of intruder and unrelated as well as related territory holders. Moreover, standard length of the intruder and the difference in standard length between both territory holders did not affect the index of relative times spent close to either related or unrelated conspecifics significantly (Table 1).

**FIGURE 3:**
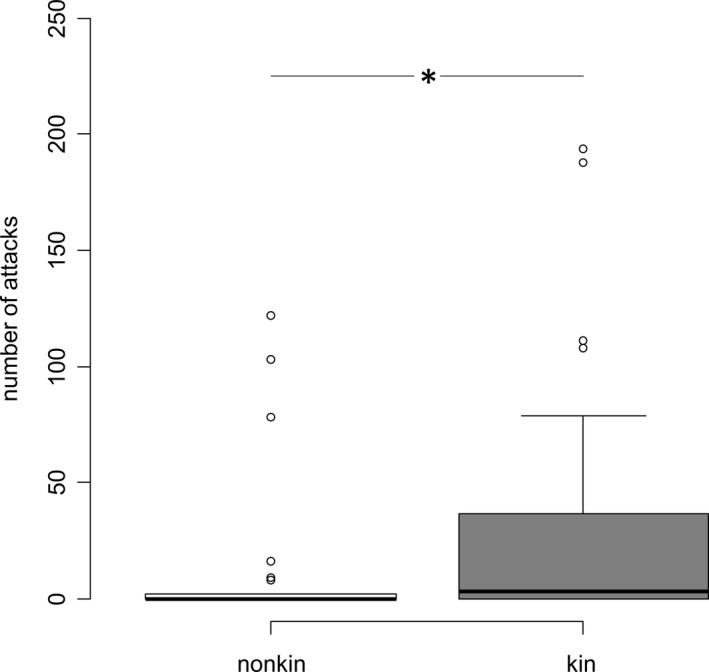
Shown are the number of attacks from the intruder directed to nonkin and kin. Given are medians, first, and third quartiles. Whiskers represent the last data point within the 1.5 times interquartile ranges (IQR). Circles show data points outside the IQR. *p < .05

Intruders were more aggressive against kin than nonkin (paired Wilcoxon‐Test: *N*_kin _= 40, *N*_nonkin_ = 40, *V* = 133.5, *p* = .043, Figure 3) whereas territory holders aggression directed to kin or nonkin did not differ significantly (paired Wilcoxon‐Test: *N*_kin _= 40, *N*_nonkin_ = 40, *V* = 250, *p* = .422).

## 5 DISCUSSION

Related intruders triggered more approaches and more time spent close to the intruder zone in territory holders than unrelated competitors. Also, intruder males attacked brothers more often than unrelated opponents. The results indicate higher intrasexual territorial aggression among relatives. Territory holders spent increased time close to kin which potentially aims to expel the closely related conspecific from their own territory. Higher number of visits together with increased aggression of the intruders could be caused by limited spatial avoidance possibilities or may reflect the intention of taking over the territory, that is, expelling the related territory holder. Even if displacing a related conspecific has indirect fitness costs (because he loses his territory), these costs may be outweighed by not directly competing with kin for mating partners later. This could be especially true for our system, because the natural habitat of the Moliwe population of *P. taeniatus* is well structured, including a variety of breeding sites (TT, personal observation), which likely should allow the displaced individual to occupy a territory elsewhere.

Here, behavioral responses are strongly influenced by kinship, that is, kin competition. However, based on our data, it cannot be distinguished which side initiated such behaviors as individuals can communicate their aggressive motivation prior physical interaction even from distance using different modalities, for example, visual cues (Frommen, 2020).

Although the presence of closely related conspecifics has the potential to promote cooperative behavior in various taxa (West & Gardner, 2010; West et al., 2002) including fishes (reviewed in Ward & Hart, 2003), kin competition is a strong evolutionary force which can limit kin‐related, cooperative benefits (Platt & Bever, 2009; West et al., 2002) and result in kin avoidance. Furthermore, other aspects, for example, negotiation and appeasement, may even have a higher potential to cause sociality than kin selection (Quiñones, van Doorn, Pen, Weissing, & Taborsky, 2016). Accordingly, in Norway rats (*Rattus norvegicus*), relatedness can decrease cooperative behavior (Schweinfurth & Taborsky, 2018) and in the cooperatively breeding cichlid *Neolamprologus pulcher*, related helpers show less direct brood care than unrelated ones (Zöttl, Heg, Chervet, & Taborsky, 2013).

Evading kin competition was mainly observed in terms of kin‐promoted dispersal behavior (e.g., Gandon, 1999; Van Petegem et al., 2018). However, in the present study, another mechanism may have led to the increased aggression between kin. Under certain circumstances, for example, when costs of unconditional dispersal are extreme or natural barriers impede dispersal, the presence of kin may result in aggressive behavior aiming to expel potential rivals. Accordingly, in the sea anemone *Actinia equina*, relatedness causes increased aggression between competing individuals (Foster & Briffa, 2014) and kinship also increases aggression in the polyembryonic wasp *Copidosoma floridanum* (Dunn, Dunn, Strand, & Hardy, 2014).

Intrasexual kin avoidance has also been observed in *P. taeniatus* which show promoted exploration in the presence of same‐sex kin (Vitt et al., 2020) and avoid breeding caves including olfactory cues of full‐siblings (Thünken, Waltschyk, Bakker, & Kullmann, 2009). However, kinship was also shown to promote cooperation in *P. taeniatus*. Previous studies on *P. taeniatus* showed that kinship reinforces cooperative predator inspection (Hesse et al., 2015) and that juvenile individuals show a shoaling preference for kin (Hesse & Thünken, 2014; Thünken et al., 2015). However, shoaling with kin is avoided in hungry juveniles, reducing kin competition (Thünken, Hesse, & Meuthen, 2020). The results of the present study highlight the importance of plastically adjusted behavior to the presence of kin or nonkin and show that proximity of kin causes context‐dependent preference or avoidance in this species.

Individuals were able to discriminate kin despite being separated nearly their whole life and adjusted their behavior accordingly. Previous studies already confirmed phenotype matching in *P. taeniatus* of the same population used here (Thünken, Bakker, & Baldauf, 2014; Thünken et al., 2007a). Potential familiarity effects on dominance relationships, which may have been established during the first months being kept together, are unlikely as individuals were separated prior maturation and kept without contact for about 14 months.

Taken together, kinship promotes aggression and may ultimately cause dispersal in subordinate individuals under natural conditions. Costs of leaving the natal area mostly are unpredictable, and exploring novel environments is risky (Bonte et al., 2012). However, dispersal may allow beneficiaries when followed by competition against nonrelatives (Wilson et al., 1992). This would include an “altruistic” aspect of dispersal behavior which, in a theoretical modeling approach, could enhance inclusive fitness (Taylor, 1988). Thus, leaving may be beneficial when costs of kin competition exceed costs of dispersal (Gandon, 1999). As mentioned above, expelling rivals may be an alternative strategy to circumvent kin competition and costs of dispersal. Accordingly, kin avoidance by forced displacement of related conspecifics has been shown for gray jays (*Perisoreus canadensis*) (Strickland, 1991). We showed kin discrimination in an intrasexual, territorial context in adult male *P. taeniatus* and disclosed the impact of intrasexual kin competition on territorial defense, representing a fitness‐relevant behavior. Thus, *P. taeniatus* is capable of plastically adjusting kin‐related behavior to different contexts which resulted in promoted aggression potentially followed by expulsion or emigration of related rivals. This study elucidates the importance of kinship in an intrasexual social conflict situation which ultimately may affect individual's fitness.

## CONFLICT OF INTEREST

We declare that we have no competing interests.

## AUTHOR CONTRIBUTION

**Simon Vitt:** Conceptualization (equal); Data curation (lead); Formal analysis (lead); Investigation (lead); Methodology (equal); Supervision (equal); Validation (equal); Visualization (lead); Writing‐original draft (lead); Writing‐review & editing (equal). **Jenny Hiller:** Formal analysis (equal); Investigation (supporting); Methodology (equal); Writing‐review & editing (supporting). **Timo Thünken:** Conceptualization (equal); Data curation (supporting); Formal analysis (supporting); Funding acquisition (lead); Investigation (supporting); Methodology (supporting); Project administration (lead); Supervision (lead); Validation (equal); Writing‐original draft (supporting); Writing‐review & editing (equal).

## Data Availability

Analyses reported in this article can be reproduced using the data stored in the Dryad repository: https://doi.org/10.5061/dryad.gxd2547jc.
